# Spectroscopy-multimodal data fusion empowers smart food quality analysis: Challenges and prospects

**DOI:** 10.1016/j.fochx.2026.103939

**Published:** 2026-04-30

**Authors:** Zhanming Li, Wenxuan Deng, Jing Zhao, Yan Kong

**Affiliations:** a School of Grain Science and Technology, Jiangsu University of Science and Technology, Zhenjiang, Jiangsu, 212100, China; b National University of Singapore (Suzhou) Research Institute, 377 Lin Quan Street, Suzhou Industrial Park, Suzhou, Jiangsu, 215123, China

**Keywords:** Food quality, Multimodal data fusion, Lightweight, Food traceability, Adulteration

## Abstract

Food quality assessment faces challenges in detecting complex samples, with single spectroscopic techniques limited in precision and applicability. Multimodal data fusion, by combining different spectroscopic techniques with non-spectroscopic data, significantly enhances the accuracy and comprehensiveness of detection. Low-, mid-, and high-level data fusion strategies offer substantial advantages in information integration and model optimization, addressing the shortcomings of single spectroscopic methods in complex food sample analysis. Multimodal data fusion has shown great potential in food adulteration detection, geographical traceability, and quality assessment. However, current technologies still face challenges such as data consistency, equipment integration, and model generalization. Future research will focus on lightweight equipment development, deep learning integration, standardization, and optimized data processing workflows to advance the development and application of food quality detection technologies.

## Highlights

• Single-modal spectroscopy limits high-speed, multi-index food detection
• Multimodal fusion enhances accuracy and comprehensiveness in food analysis
• Low-, mid-, and high-level fusion improve detection across key applications
• Future work focuses on lightweight systems, model adaptability, and standardization

## 1 Introduction

Food quality and safety assessment is central to public health protection and market regulation, covering authenticity verification, adulteration detection, traceability, and contaminant monitoring. While conventional analytical techniques such as gas chromatography–mass spectrometry (GC–MS) and high-performance liquid chromatography (HPLC) provide high analytical accuracy, their labor-intensive workflows, long analysis times, and destructive sample preparation limit their applicability for rapid, large-scale, and on-line food monitoring (Feng et al., 2022; Sugiura et al., 2023). These constraints have driven growing interest in spectroscopic technologies, including near- and mid-infrared spectroscopy (NIRS and MIRS), Raman spectroscopy (RS), and hyperspectral imaging (HSI), which enable non-destructive, rapid, and information-rich analysis (Fang et al., 2026; Lacerda et al., 2025; Li et al., 2024; Lu, Qin, et al., 2026).

Models based on a single spectroscopic technique have therefore been widely applied in food quality assessment due to their methodological maturity, relatively low modeling cost, and ease of implementation (Yu, Chai, Li, et al., 2025). The homogeneous structure of single-modal spectral data allows direct model construction without complex cross-modal preprocessing, and reliable performance can often be achieved in well-defined application scenarios. RS can exhibit high sensitivity to molecular structures and has been successfully used for trace detection of pesticide residues (Mikac et al., 2021) while HSI technique enables simultaneous acquisition of spatial and spectral information, facilitating correlation analysis between external defects and internal quality attributes of foods (Zhang et al., 2020).

However, as food systems become increasingly complex, the limitations of single spectroscopic modalities have become more evident. Single-modal data inherently capture only restricted information dimensions, resulting in limited robustness and reduced adaptability across different food matrices and processing conditions. For multi-component or structurally heterogeneous foods (such as nut butters or meat products), models developed from a single spectral source often exhibit poor generalization performance (Geng et al., 2019; Ghosh & Datta, 2023). In addition, single-modal approaches struggle to meet the growing demand for simultaneous multi-index detection in modern food quality monitoring.

**Fig. 1:**
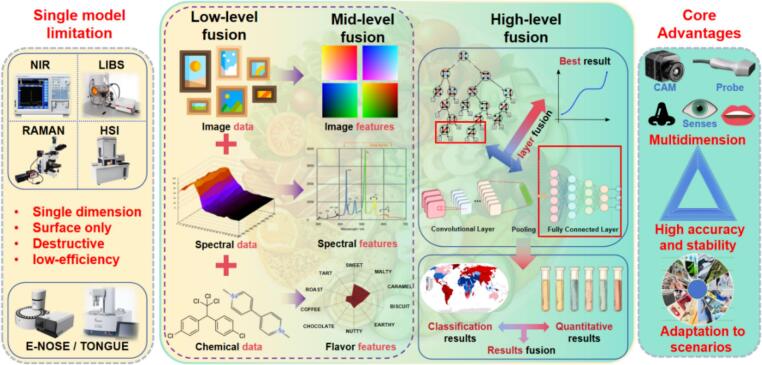
Scheme of the applications of spectral-based multimodal data fusion technology in food quality analysis.

To overcome these challenges, multimodal data fusion has emerged as a promising strategy by integrating complementary information from multiple spectroscopic techniques or by combining spectroscopic data with non-spectroscopic physicochemical indicators. By exploiting information complementarity, multimodal models can significantly improve prediction accuracy, robustness, and scenario adaptability (Revilla et al., 2019; Wu et al., 2023). NIRS data fused with physicochemical parameters (protein, fat, moisture, and acidity) has been employed to develop prediction models using random forest (RF) and partial least squares–support vector regression (PLS-SVR), with the RF model achieving correlation coefficients above 0.96 and a maximum residual predictive deviation (RPD) of 20.14 (Peng et al., 2025). These findings highlight a growing consensus that multimodal modeling provides a more robust solution for complex food quality assessment than reliance on any single spectroscopic modality. This review focused on the application of multimodal data analysis based on spectroscopic techniques in food quality evaluation (Fig. 1), introduced the inherent differences, applicable scenarios, and practical selection criteria of different fusion strategies, outlined the main challenges faced by spectral data fusion, and proposed a prospective research framework to provide new perspectives for future studies, aiming to advance the development of food spectroscopic techniques and multimodal data fusion modeling.

## 2 Food spectral-based data fusion technology

In the food field, for key demands such as geographical traceability, quality assessment and processing detection, single spectroscopic techniques often suffer from insufficient accuracy, limited comprehensiveness or narrow application scenarios (Guo, Lin, Chen, et al., 2025). To solve these problems, research on low-level data fusion of multimodal food spectral data has gradually emerged. Most studies integrate different spectral modalities or spectral data with other information combined with machine learning algorithms, improving analytical performance via multi-source information synergy and providing new ideas for technological innovations in food-related fields (Strani et al., 2024).

**Fig. 2:**
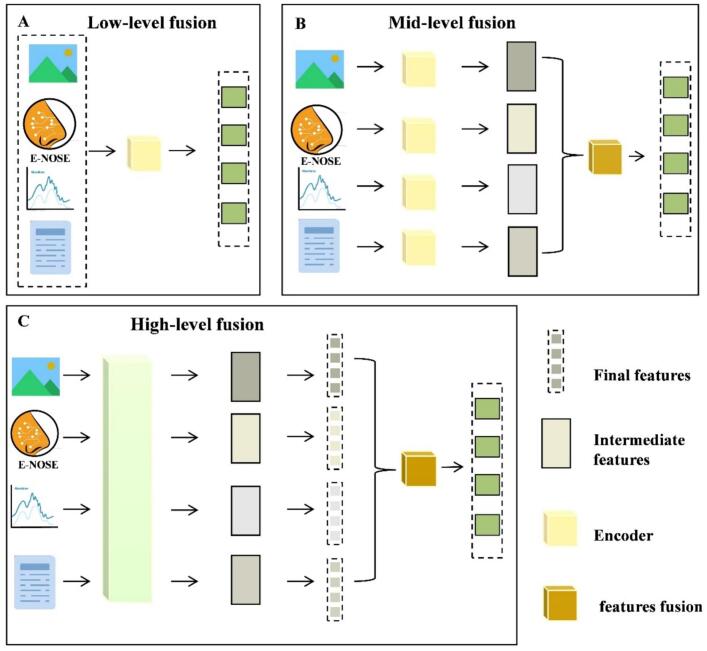
Scheme of multimodal fusion methods, including (A) low-level data fusion, (B) mid-level data fusion, and (C) high-level data fusion. Adapted from **(**Xiao et al., 2025**)** with permission.

Multimodal data analysis refers to the integration of two or more types of heterogeneous data (e.g., different spectroscopic techniques or spectroscopy and non-spectroscopy data) for modeling. Multimodal data modeling is not merely a simple data concatenation, but involves effective integration at the low level (data level), middle level (feature level) or high level (decision level) (Fig. 2). In recent years, multimodal data fusion methods based on food spectroscopic techniques have garnered extensive attention and found widespread application in the field of food quality analysis (Saklani et al., 2025; Yokoya et al., 2017). Additionally, deep learning methods exhibit excellent performance in achieving cross-modal feature alignment and information extraction (Shokraei Fard et al., 2022).

In multimodal food spectroscopy data analysis, different fusion strategies exhibit significant differences in information integration logic, feature utilization efficiency, and computational complexity. These differences directly influence model performance and the applicability of each strategy. Low-level, mid-level, and high-level data fusion correspond to different modeling paradigms, each involving a trade-off between information retention, feature compression, and decision-level collaboration (Pawłowski et al., 2023). The core distinction lies in how to balance information integrity, noise suppression, and model complexity, ultimately determining the strengths and limitations of each strategy. Therefore, in practical applications, the selection of fusion strategies should not only consider data characteristics and task requirements, but also take into account validation settings and modality differences. A rational and context-aware selection of fusion strategies is essential to achieve efficiency, accuracy, and generalizability in multimodal food spectroscopy analysis (Guarrasi et al., 2025).

### 2.1 Low-level data fusion

Low-level data fusion involves the concatenation or joint modeling of raw data, maximizing the retention of original signal features, which is beneficial for capturing the direct complementary relationships between modalities. Therefore, this strategy is more suitable for scenarios with lower data dimensions, higher signal-to-noise ratios, and stronger correlations between modalities (Guo et al., 2024). However, due to the lack of effective information selection mechanisms, low-level data fusion is prone to introducing redundant variables and amplifying noise interference, which increases the computational burden during model training and reduces prediction accuracy.

When sample sizes are limited or data quality is uneven, the accumulation of high-dimensional redundancy and noise often further compromises the robustness and generalizability of the model. From a validation perspective, the advantages of low-level data fusion are typically observed under conditions of large sample sizes, high-quality data, and low noise (Liu, Yin, et al., 2025). Conversely, when sample sizes are small or data quality is inconsistent, the performance of low-level data fusion may be adversely affected, and external validation or cross-domain validation frequently reveals fluctuations in predictive accuracy. Therefore, the benefits of low-level data fusion are generally highly context-dependent, with its applicability largely determined by the scale of the task and the quality of the data. Under different validation scenarios, particularly in cross-batch or cross-device evaluations, low-level data fusion is more susceptible to noise, resulting in less stable performance (Jiao et al., 2024).

**Fig. 3:**
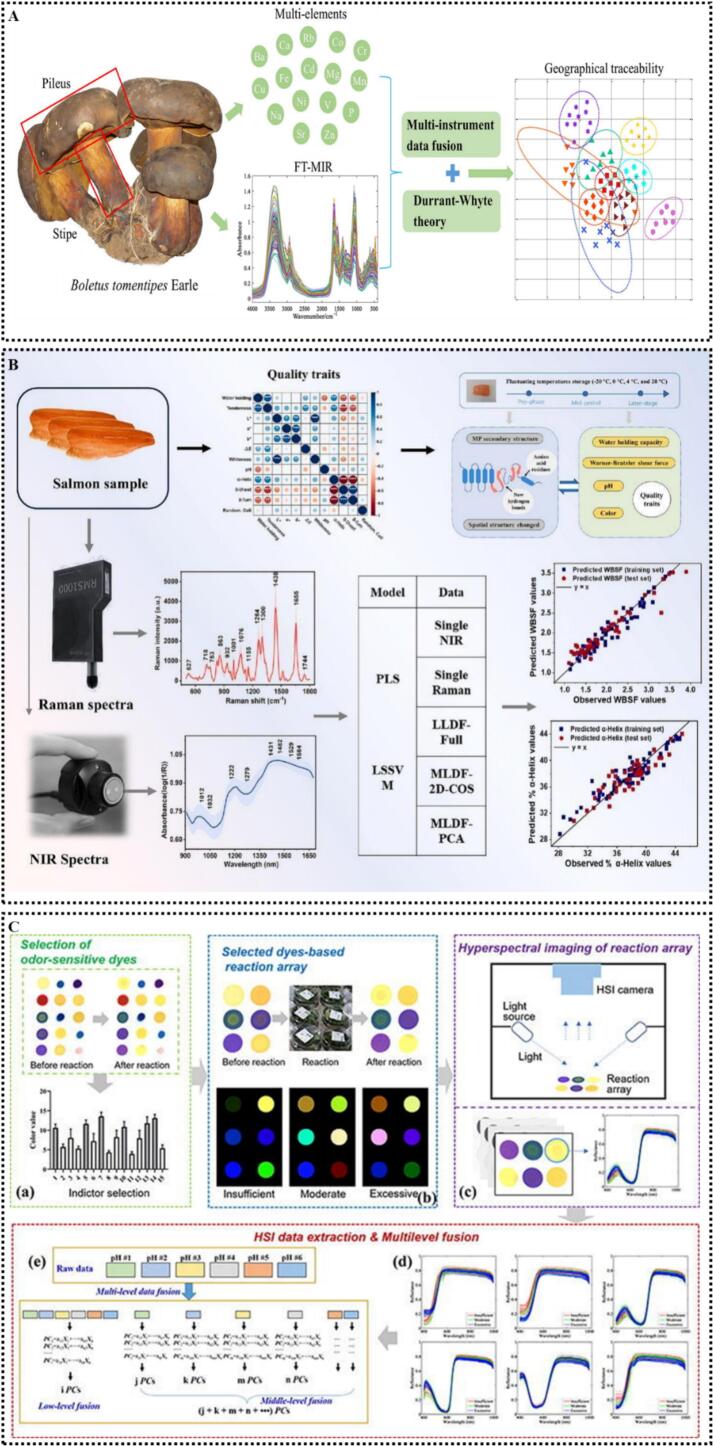
(A) Collaborative strategy for geographical traceability of wild Boletus based on data fusion analysis; (B) improving the accuracy of salmon quality assessment via multi-source molecular spectroscopy data fusion and machine learning; (C) determination of black tea shrinkage degree by combining colorimetric sensor array and HSI, (a) indicator selection, (b) reaction array, (c) HSI and spectrum, (d) raw data, and (e) data fusion. Cited from (Guo, Lin, Chen, et al., 2025; Li & Wang, 2018; Wang et al., 2022) with permission.

In a study on the geographical traceability of wild Boletus, targeting *Boletus tomentipes* from 10 sampling sites in Yunnan, China, researchers collected Fourier transform MIRS and 16 element contents detected by inductively coupled plasma-atomic emission spectroscopy, then constructed support vector machine (SVM) and RF classification models via data fusion. The results showed that multimodal low-level data fusion significantly outperformed single-modal analysis; the 10-fold cross-validation accuracy reached 94.23%, and the grid search SVM model even achieved 100% accuracy in external validation, providing an efficient and reliable technical pathway for the geographical traceability of wild fungi (Li & Wang, 2018) **(**Fig. 3A**)**.

In terms of animal food quality detection, researchers have conducted studies on the quality changes of salmon stored at different temperatures: they performed low-level data fusion of NIRS and RS data, and constructed Warner-Bratzler shear force prediction models combined with PLS and least-squares SVM (LS-SVM). The LS-SVM model based on fused data performed the best, significantly outperforming single-spectrum models. Moreover, a temperature compensation strategy enhanced the model's robustness under temperature fluctuation scenarios, enabling rapid and non-destructive detection of salmon quality (Guo, Lin, Chen, et al., 2025) **(**Fig. 3B**)**. Furthermore, a study constructed an LS-SVM discriminant model for black tea withering degree evaluation based on the low-level data fusion of colorimetric sensor array and hyperspectral data; the calibration set accuracy reached 93.75% and the prediction set accuracy 90%, which was significantly superior to modeling with single pH indicator spectra (maximum 80%) (Wang et al., 2022) **(**Fig. 3C**)**.

Low-level data fusion of spectroscopy combined with other data has provided effective technical pathways for demands in the food field, such as geographical traceability, rapid quality assessment, and intelligent processing quality detection (Table 1). Its characteristic of improving analytical accuracy and applicability through multi-source information synergy has not only addressed the shortcomings of single techniques but also offered new methods for the food industry to move toward precise and efficient detection (Firmani et al., 2020).

**Table 1:** Applications and performances of different fusion levels of spectral and non-spectral technologies in food detection.

**Category**	**Spectral technology**	**Non-spectral Data**	**Fusion level**	**Performance**	**References**
Origin traceability of *Cordyceps sinensis*	NIRS, HSI	Color features, texture features	Mid-, high-level	Classification Accuracy: 88.89%	(Ren et al., 2025)
Geographic traceability of Chinese yam	NIRS, MIRS, μRS	None	Mid-level	Traceability Accuracy: 100%	(Gao et al., 2024)
Geographic traceability of *Poria cocos*	FTIR	UFLC data	Low-level	FTIR-LC fusion model: 100% accuracy for both calibration and validation sets; R^2^(cum) = 0.9599, Q^2^(cum) = 0.7917	(Wang et al., 2019)
Geographic traceability of *Panax notoginseng*	FT-MIRS, NIRS	None	Low-, mid-, high-level	Low level: Calibration set 93.7%, Validation set 95.3% Mid-level: Calibration set 97.7%, Validation set 97.7% High level: Calibration set 98.9%–99.4%, Validation set 100%	(Li et al., 2018)
Origin identification of Honeysuckle	NIRS, MIRS	HPLC data	High-level	Origin identification: Fusion model prediction set accuracy 95.5%, Kappa = 0.910, log-loss = 0.347, outperforming single NIR (90.9%) and MIR (93.2%) models	(Hao et al., 2024)
Geographic traceability of extra virgin olive oil	FT-RS, FS	None	Low-, high-level	Low-level data fusion: Test set sensitivity 73–93%, specificity ≥93% High-level data fusion: Better performance than low level, validation set accuracy 93%–97%	(Fort et al., 2021)
Species identification and component prediction of *Curcuma zedoaria*	UV, FT-NIRS, FT-IR	HPLC data, sample morphology data	Low-, mid-level	Species identification accuracy: 100%; Content prediction RPD up to 78.32	(Ren et al., 2025)
Soybean variety identification	Vis-NIRS-HSI	RGB feature data	Low-, mid-, high-level	High-level data fusion (optimal): Validation set accuracy 93.13%, F1-score = 93.70%, AUC > 0.98 Low-level data fusion: Validation set accuracy 91.88%, F1-score = 92.29% Mid-level data fusion: Validation set accuracy 86.25%, F1-score = 87.41%	(Gao et al., 2024)
Rapid identification of fish species	LIBS, RS	None	Low-, mid-, high-level	1. Low-level data fusion: Classification accuracy 98.2%, AUC = 0.9799 2. Mid-level data fusion: Accuracy 96.0%, variables reduced from 9213 to 200, computational efficiency improved (0.44 h vs 20.05 h), enhanced model interpretability 3. High-level data fusion: Accuracy 95.8%, AUC = 0.9769	(Ren et al., 2023)
Adulteration classification and quantification of saffron	RS	Thin-layer chromatography imaging	Mid-level	Classification validation set accuracy: 99.20%; Quantification maximum R^2^ = 0.990, minimum RMSEP = 3.11	(Dai et al., 2023)
Adulteration detection of camellia oil	NIRS	Smartphone image/video data	Mid-, high-level	NIR + Image/Video fusion accuracy: 96.30%	(Deng et al., 2025)
Mutton freshness detection	HSI	EN Data	Mid-level	Training set RMSET = 3.027 mg / 100 g, R^2^ = 0.922 Prediction set RMSEP = 3.039 mg / 100 g, R^2^ = 0.920, RPD = 3.59	(Liu et al., 2022)
Red meat quality detection	RS, MIRS	None	High-level	pH prediction: High-level data fusion (optimal), validation set R^2^p = 0.73, RMSEP = 0.22, NRMSEP = 12.9%, outperforming single Raman or FTIR	(Robert et al., 2021)
Lipid oxidation detection of salmon	NIRS, RS	TBARS values	Low-, mid-, high-level	CNN model R^2^p = 0.866, RMSEP = 0.103 mg MDA/kg	(Guo, Lin, Feng, et al., 2025)
Fungal contamination detection of soybeans	Vis-NIRS, SWIR	None	Low-, mid-level	1. Low-level data fusion 1D-CNN: Validation set accuracy 97.52%, Precision = 97.55%, Recall = 97.52%, F1-score = 97.52% 2. Mid-level data fusion 1D-CNN: Accuracy 97.85%, F1-score = 97.86%; Transformer model accuracy 95.71%	(Shao et al., 2025)
Tomato ripeness assessment	Vis-NIRS	RGB image, tactile data	Mid-level	Classification accuracy: 99.4%; Accuracy for uneven internal-external ripeness: 94.4%	(Liu et al., 2024)
Ripeness identification of preserved eggs	RH, TH	EN data	Mid-level	PCA-LDA model (EN + RH + TH fusion): Training accuracy 98.89%, prediction accuracy 95.56%	(Ren et al., 2021)
Pu'er tea year identification	THz, RS	None	Low-, mid-level	1. Mid-level data fusion (optimal): Validation set accuracy 98.95%, F1-score = 0.9896 2. Low-level data fusion: Accuracy 94.79%, F1-score = 0.9482	(Zhang et al., 2025)

Notes: μRS, micro-Raman spectroscopy; FTIR, Fourier transform infrared spectroscopy; UV, ultraviolet spectroscopy; FS, fluorescence spectroscopy; FT-IR, Fourier transform-infrared spectroscopy; Vis-NIRS-HSI, visible-near infrared hyperspectral imaging; LIBS, laser-induced breakdown spectroscopy; SWIR, short-wave infrared spectroscopy; UFLC, ultra-fast liquid chromatography; RH, reflectance hyperspectral; TH, transmittance hyperspectral; THz, terahertz spectroscopy; EN, electronic nose; HPLC, high-performance liquid chromatography; FT-RS, Fourier transform-Raman spectroscopy; TBARS, thiobarbituric acid reactive substances; CNN, convolutional neural network; R^2^, coefficient of determination; RMSET, root mean square error of training.

### 2.2 Mid-level data fusion

As an intermediate-level data fusion approach built on low-level data fusion, feature extraction is performed first. By effectively integrating multiple data sources, this method can better capture the complementarity among various spectral signals, thereby significantly improving prediction accuracy and robustness **(**Table 1**)**. It is particularly well-suited for the refined analysis and efficient prediction of complex samples (Legner et al., 2020).

Mid-level data fusion involves the extraction and selection of features from each modality before fusion, enabling the compression of data dimensions while retaining key information to achieve deep collaboration among multiple data sources (Silva et al., 2025). This dimensionality reduction followed by fusion strategy is more adaptable when dealing with high-dimensional, complex, or noisy multimodal data, and is particularly suitable for food quality detection and adulteration analysis using multispectral, hyperspectral, and chromatography-mass spectrometry data integration (Xiao et al., 2025). However, its performance is highly dependent on the feature extraction methods and feature selection strategies used, and inappropriate feature engineering may lead to the loss of critical information or introduce the risk of overfitting. Moreover, mid-level data fusion entails higher complexity in data preprocessing, parameter optimization, and model construction, placing greater demands on computational resources and algorithmic design capabilities (Gao et al., 2024; Lu, Zhao, et al., 2026). In validation practice, mid-level data fusion often maintains more stable performance in multimodal validation and independent test sets, particularly in tasks with large sample sizes and strong modality redundancy, where it effectively mitigates the negative impact of redundant features and strengthens discriminative capability (Jiao et al., 2024).

**Fig. 4:**
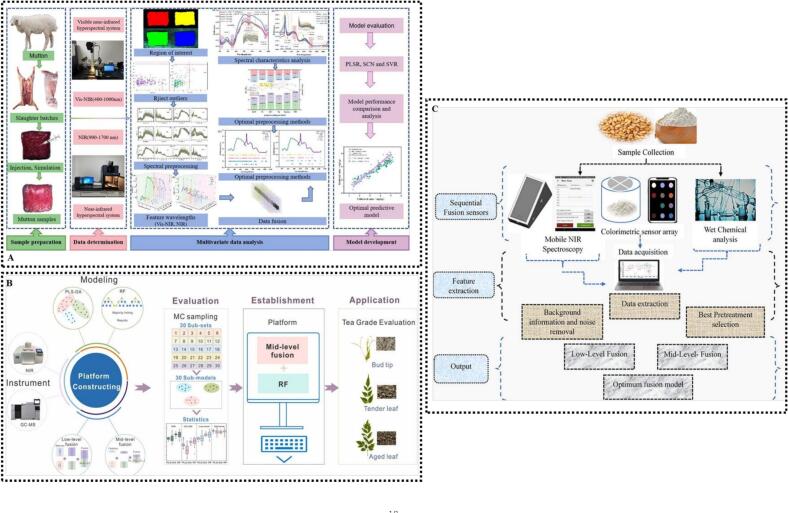
(A) Fusion of Vis-NIRS and HSI techniques with data fusion strategy for predicting norfloxacin residues in mutton; (B) discrimination of vine tea grades based on NIRS and GC–MS techniques; (C) quantitative analysis of free fatty acids in wheat using fusion strategy of CSA and portable NIRS combined with chemometrics. Cited from (Feng et al., 2024; Li et al., 2023; Zareef et al., 2023) with permission.

In a study on norfloxacin residues in mutton, Visible-near infrared spectroscopy (Vis-NIRS) was combined with an HSI system. The mid-level data fusion strategy integrated with a stochastic configuration network model achieved a prediction set coefficient of determination for prediction set (R^2^p) of 0.9312 and a root mean square error of prediction (RMSEP) of 0.3316, significantly improving detection accuracy (Feng et al., 2024) (Fig. 4A). Similarly, multimodal fusion of ICP-MS, isotope ratio MS, and UPLC/IM-QTOF-MS data enabled accurate wine origin traceability, with an artificial neural network achieving 98.7% classification accuracy under both ESI+ and ESI− modes (Su, Wang, et al., 2025). In another study on the grade discrimination of vine tea, mid-level data fusion was adopted to integrate NIRS and GC–MS data, combined with a RF model for modeling. Validated via Monte Carlo methods, the model achieved an accuracy of 92.38%, providing an efficient approach for vine tea quality assessment (Li et al., 2023) **(**Fig. 4B**)**. These studies have shown that mid-level data fusion has advantages in integrating multi-source data and improving model performance, offering an efficient technical pathway particularly for complex sample identification and food origin traceability.

In the quantitative analysis of free fatty acid content in wheat flour, low-level data fusion and mid-level data fusion strategies were employed, combining a colorimetric sensor array (CSA) and NIRS with a PLS model. The results indicated that mid-level data fusion outperformed low-level data fusion, providing an effective solution for the rapid quality detection of wheat flour (Zareef et al., 2023) **(**Fig. 4C**)**. Additionally, in the adulteration detection of Ganoderma lucidum spore powder, NIRS and HSI techniques were combined, with PLS-DA and PLSR models used for analysis. Low-level data fusion directly concatenated the two types of spectral data, resulting in an adulteration detection accuracy of 92.86%, a prediction set R^2^_p_ of 98.10%, and an RMSEP of 5.30%. In contrast, mid-level data fusion fused data after feature selection and applied an RF model for adulteration detection; the model achieved 100% accuracy, precision, recall, and F1-score in both the training and prediction sets. These results have shown that mid-level data fusion significantly outperformed low-level data fusion, with overall performance also exceeding that of single-spectral techniques and low-level data fusion (Jiang, Zhong, et al., 2023).

In summary, as a critical strategy in multimodal data fusion, mid-level data fusion has established a stronger information bridge between feature extraction and modeling, enabling deep synergistic enhancement across multi-source data. Its superior performance in complex food systems has not only significantly improved the accuracy and robustness of non-destructive testing but also provided a more intelligent technical pathway for applications such as food and drug residue monitoring, origin traceability, and quality evaluation.

### 2.3 High-level data fusion

High-level data fusion has achieved deep fusion and collaborative modeling of multimodal information at the decision level, which can fully leverage the characteristic advantages of different spectroscopic techniques (Table 1), effectively enhance the robustness and generalization ability of models, and demonstrate higher detection accuracy and stability (Le et al., 2025). High-level data fusion integrates the outputs of individual modality-specific models at the decision level, using voting, weighting, or consensus strategies to achieve multi-model collaboration and fully exploit the complementary advantages among modalities. This strategy is particularly suitable for complex applications where data sources are highly heterogeneous and single modalities cannot comprehensively characterize sample features, such as the integration of spectral and imaging data for variety identification or adulteration detection. Because each modality is modeled independently before integration, high-level data fusion can mitigate biases from individual modalities to a certain extent, thereby enhancing overall predictive robustness and generalizability (Gu et al., 2025).

From a validation standpoint, high-level data fusion can effectively address the challenges posed by multi-source data. It is particularly advantageous in multi-modal, multi-batch, or cross-device testing scenarios, where integrating information from different modalities enhances both robustness and consistency (Rong et al., 2024). Nevertheless, because this approach requires larger sample sizes and greater computational resources, its advantages are most pronounced in environments with high data heterogeneity, complex tasks, and sufficient computational capacity. Under conditions of limited samples or restricted computational resources, the performance benefits of high-level data fusion may be constrained, and its applicability in practical settings is relatively limited due to the higher requirements for system integration and maintenance (Deng et al., 2026; Gu et al., 2025).

**Fig. 5:**
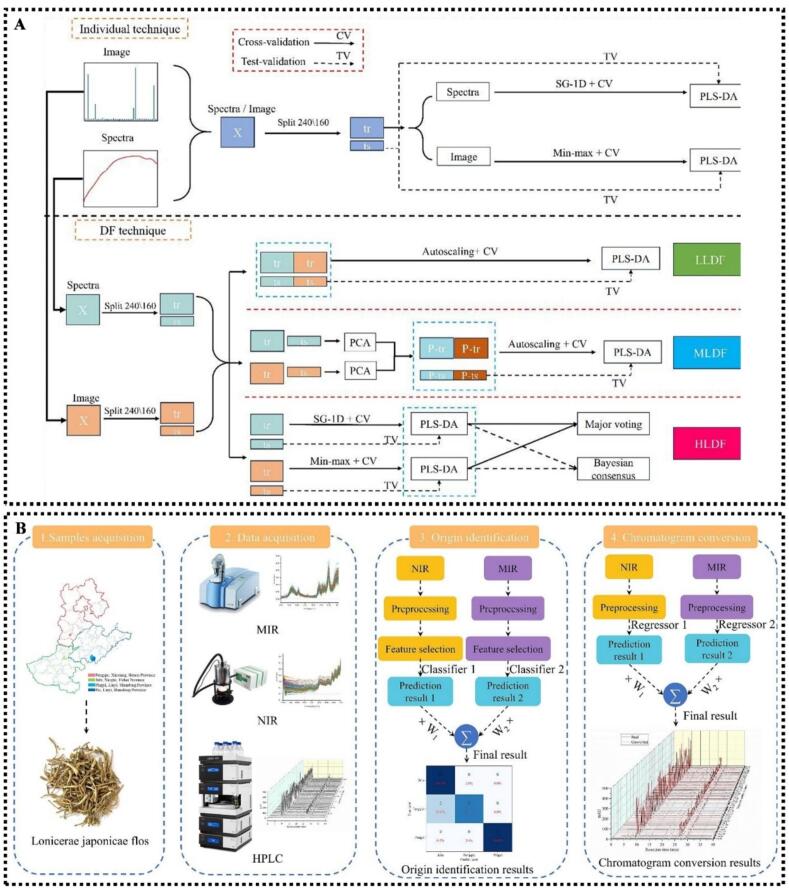
(A) Multi-level data fusion strategy for soybean seed cultivar identification based on spectral and image information; (B) fusion of NIRS and MIRS data for rapid geographical origin identification of honeysuckle. Cited form (Gao et al., 2024; Hao et al., 2024) with permission.

A study on six French extra virgin olive oil varieties compared low-, mid-, and high-level data fusion of NIRS and MIRS data for variety identification. High-level data fusion, leveraging spectral complementarity through majority voting, outperformed other strategies, achieving AUC values of 1.00 for Cailletier and 0.97 for Aglandau with improved model balance and generalization (Maléchaux et al., 2020). Similarly, in the variety identification of soybean seeds, a spectral-image multi-level fusion strategy based on HSI was adopted to compare the fusion effects of low-level data fusion (accuracy: 91.88%) and mid-level data fusion (accuracy: 86.25%). Results indicated that high-level data fusion (Bayesian consensus) performed the best, achieving a test set accuracy of 93.13%, an F1-score of 93.70% and an AUC > 0.98, with the lowest degree of overfitting. This strategy effectively integrated complementary information, providing an efficient and reliable solution for seed variety identification (Gao et al., 2024) **(**Fig. 5A**)**.

Likewise, in the quantitative analysis of high-fructose glucose syrup adulteration in honey, a study systematically evaluated the performance of three fusion strategies combined with PLS models based on MIRS and Raman spectra. The results showed that high-level data fusion exhibited stronger stability and fault tolerance compared with low and mid-level data fusion (Li et al., 2020). This strategy effectively avoided the redundant interference in low-level data fusion and the feature omission in mid-level data fusion, fully unleashing the chemical complementary advantages of the two spectroscopic techniques. Similarly, in the study on geographical origin identification and quality evaluation of honeysuckle, a high-level data fusion strategy was adopted based on NIRS and MIRS spectral data. When soft voting was used for origin identification, the model achieved a prediction set accuracy of 95.5%, a log-loss of 0.347 and a Kappa value of 0.910, which were significantly superior to those of single NIRS (90.9%, 0.372, 0.817) and single MIRS (93.2%, 0.351, 0.863). This high-level data fusion strategy effectively integrated dual-spectral information, significantly improving model performance and accuracy (Hao et al., 2024) **(**Fig. 5B**)**.

As above, high-level data fusion has realized deep collaboration of multimodal information at the decision level, which can fully utilize the complementarity of different spectroscopic techniques and significantly enhance the robustness and generalization ability of models, offering more intelligent alternatives for modeling complex food systems (Xiao et al., 2025).

### 2.4 Deep learning-based multimodal data fusion

In traditional multimodal data fusion studies, low-level, mid-level, and high-level data fusion strategies commonly rely on manually designed feature extraction and predefined rules. These approaches achieve information integration through feature concatenation, weighting, or decision-level aggregation. Although they can improve model performance under certain conditions, their fundamental limitation lies in the strong dependence on handcrafted features and prior rules (Hangloo & Arora, 2025). As a result, their performance is constrained when handling high-dimensional, complex, or nonlinear inter-modal relationships, and they are more susceptible to noise interference. Specifically, these methods are generally incapable of automatically extracting latent high-level features from data and often struggle to capture complex nonlinear correlations between modalities, thereby limiting their effectiveness in multimodal data analysis.

In contrast, modern deep learning approaches enable end-to-end modeling and automated feature learning, allowing adaptive extraction of informative features from multimodal data and facilitating deeper information integration (Zhang, Zhou, et al., 2022). These methods no longer rely on manually designed features but instead construct data representations through multi-level and multi-scale representation learning. They demonstrate significant advantages in multimodal representation learning and cross-modal alignment. In cross-modal alignment, deep learning models leverage attention mechanisms or Transformer-based frameworks to dynamically learn relationships between modalities, thereby achieving automatic alignment and avoiding the limitations of explicit annotations or predefined mappings required in traditional approaches (Lou et al., 2024; Xu et al., 2023). Through these mechanisms, models can capture deeper inter-modal relationships at higher levels and perform more effective information fusion, ultimately improving task performance.

Within Transformer-based frameworks, self-attention mechanisms with deep adaptive weighting not only handle modality heterogeneity but also enable multi-level interactions across modalities (Khan et al., 2025). This addresses the issues of feature redundancy and information loss commonly associated with traditional concatenation or weighting strategies (Wang et al., 2024). Furthermore, attention mechanisms allow models to dynamically focus on the most relevant inter-modal interactions, enabling more refined multimodal fusion. Compared with conventional approaches, modern deep learning models can reduce reliance on expert knowledge while enhancing generalizability to handle complex data (Tan et al., 2024).

Overall, continuous advances in deep learning for multimodal representation learning, cross-modal alignment, and Transformer-based fusion frameworks are driving a paradigm shift from traditional feature engineering-based strategies to automated, multi-level deep learning approaches. Particularly in complex tasks such as food spectroscopy data analysis, deep learning methods provide more flexible and efficient solutions, demonstrating greater application potential and improved predictive accuracy.

## 3 Current challenges and future prospects

### 3.1 Current challenges

In multisource data fusion modeling, Low-, Mid-, and High-level data fusion strategies have been widely applied to improve the accuracy and efficiency of food quality assessment. Low-level data fusion has typically focused on simple data merging. Mid-level data fusion has addressed structural differences between heterogeneous modal data to a certain extent. High-level data fusion has given full play to the complementary advantages of each modality through the deep integration of multimodal information. Moreover, multisource data fusion modeling using different spectroscopic techniques or integrating spectroscopic and non-spectroscopic data has helped enhance the performance of food quality assessment. However, challenges have emerged in multimodal data modeling, particularly the significant structural differences across various modal data types (Dang et al., 2025; Su, Shen, et al., 2025). Spectroscopic and non-spectroscopic data have exhibited distinct structural disparities, which have necessitated complex preprocessing prior to fusion, increasing research difficulties and compromising the accuracy and generalization ability of models. The contradiction between high-dimensional data generated by food spectroscopic techniques and computational costs, as well as the imbalance between modal weight allocation and information redundancy, have posed enormous challenges for multimodal data fusion.

#### 3.1.1 Contradiction between high dimensionality and computational cost

In food quality analysis, with the continuous increase in data dimensionality, the high-dimensionality issue has become one of the major challenges for multimodal data fusion. Spectral data have typically contained hundreds or even thousands of bands, which have provided abundant information for models (Zhang, Zhang, et al., 2022). However, the increased dimensionality tends to include a large number of redundant and irrelevant features into the data, which have not only increased computational complexity but also potentially led to the curse of dimensionality, making the model training and optimization processes more difficult (Yang et al., 2022). In addition, excessively high dimensionality has also been likely to cause overfitting, thereby compromising the generalization ability and stability of models.

At the same time, the rise in computational cost has become an urgent problem to be solved. High-dimensional data have required more computational resources for processing and analysis; especially when complex models such as machine learning are adopted, the training process has needed substantial time and computing power (Li & Zhang, 2022). With the increase in model complexity, particularly in multimodal data fusion applications, the computational burden has also increased accordingly, which has restricted the deployment and real-time performance of models. To address this challenge, researchers have been exploring dimensionality reduction techniques, feature selection methods and efficient optimization algorithms to reduce computational costs and improve model operation efficiency, thus achieving high efficiency in practical applications while ensuring accuracy (Yu, Chai, Yan, et al., 2025; Zhao, Wang, et al., 2025).

#### 3.1.2 Poor consistency and anti-interference ability of spectral data

The physical state of food samples significantly affects the consistency and accuracy of spectral data. For instance, factors such as grain particle size, oil uniformity, fruit size and surface defects can all lead to variations in spectral responses. In studies on the geographical traceability of Pu-erh tea, differences in physical properties including particle size, compactness and storage conditions (e.g., airtightness) can interfere with the authentic reflection of sample chemical compositions by NIRS and RS, resulting in inconsistent or inaccurate spectral responses (Chen et al., 2025). To ensure sample consistency, Pu-erh tea samples are uniformly ground before detection, homogenized through a 40-mesh sieve, then pressed into uniform pellets under 25 MPa and stored in sealed containers. This pretreatment lays the foundation for achieving a high geographical classification accuracy of 95.05%, but it compromises the operational convenience of spectroscopic techniques.

In addition, environmental detection factors such as light intensity, temperature and humidity can interfere with spectral data, typically manifesting as spectral baseline variations accompanied by issues like noise, multiplicative effects and baseline drift. To eliminate the adverse impacts of these environmental factors on spectral data, signal preprocessing is required during the preprocessing stage of data fusion to correct interferences, including spectral baseline variations caused by environmental factors, thus ensuring the accuracy and reliability of subsequent data fusion analysis (Strani et al., 2024).

#### 3.1.3 High difficulty in cross-modal data alignment

In food spectroscopic analysis, cross-modal data alignment has become one of the major challenges currently faced. Spectral data have typically been high-dimensional, containing information across multiple bands, and such data have represented the chemical composition characteristics of food samples. However, non-spectral data have possessed distinct dimensions, sampling frequencies and structural properties, and have usually involved information such as surface morphology, color or environmental conditions. These heterogeneous data have often exhibited significant discrepancies during the alignment process, making it complex and difficult to integrate such information within a single model. For instance, spectral data have been arranged in wavelength sequences, while image data have been pixel data in two-dimensional or three-dimensional space, with substantial differences in sampling frequency and resolution between the two. The structural differences between spectral and non-spectral data have been prominent, which have necessitated complex preprocessing prior to fusion (Hong et al., 2021).

Effective alignment of these heterogeneous modal data has been crucial for improving the performance of multimodal fusion models. Inaccurate alignment has potentially led to the loss of correlations between different modalities, thereby compromising the predictive capability of the overall model (Zhu et al., 2023). If spectral information and image information have failed to be precisely aligned, models have been unable to accurately understand the relationship between the chemical composition and appearance characteristics of food, resulting in decreased accuracy of classification and detection results. Therefore, how to improve computational efficiency while ensuring the accuracy of data alignment has become one of the core issues in cross-modal data fusion (An et al., 2022). Researchers have been actively exploring efficient alignment methods to optimize the fusion process of spectral and non-spectral data, thereby enhancing the overall performance of models.

#### 3.1.4 Imbalance between modal weight allocation and information redundancy

In multimodal data fusion, the imbalance between modal weight allocation and information redundancy has become one of the critical challenges currently faced. Multimodal fusion has integrated information from different sensors or data sources. Such fusion has been able to provide richer features and stronger model expressiveness. However, the importance and relevance of information from different modalities have potentially varied in practical applications. How to reasonably allocate the weight of each modality to maximize the contribution of every modality has become the key to addressing this issue. If weight allocation has been unreasonable, the information of certain modalities may have been overemphasized while that of others neglected, thereby impairing the overall model performance (Zhang et al., 2023).

In addition, redundant information between modalities has potentially exacerbated this problem. Particularly when spectral data are highly correlated, redundant features have led to information duplication, increased computational burden, and thus limited the validity and efficiency of models (Wang et al., 2025). Actively exploring novel weighting methods, dimensionality reduction techniques, and optimization algorithms has been crucial for improving the performance and applicability of multimodal data fusion models (Guo, Lin, Feng, et al., 2025). Resolving the imbalance between modal weight allocation and information redundancy has become one of the key bottlenecks restricting the widespread application of multimodal data fusion technology in the field of food analysis.

#### 3.1.5 Poor model interpretability and regulatory compatibility

Although advanced models such as deep learning excel in detection accuracy and automation, their interpretability and regulatory compatibility remain pressing challenges to be addressed in food analysis. Many deep neural networks and ensemble learning models are often regarded as black boxes; their decision-making processes are difficult to intuitively understand and trace, and they cannot meet regulatory requirements for transparency and auditability, especially in high-risk fields like food safety detection. Currently, many models lack compliance design aligned with food safety regulations, which hinders their ability to pass compliance inspections or audits in practical applications (Shao et al., 2025).

In multimodal fusion models (e.g., CNN models combining NIR and smartphone images), some achieve high detection accuracy, but their opaque decision-making processes fail to clearly explain how a specific spectral band and visual features jointly determine the final detection results. This issue has become a major barrier to the practical regulatory application of multimodal fusion models (Deng et al., 2025). Food regulatory authorities require detection results to be traceable and verifiable, yet the ambiguous decision logic of these models makes them difficult to pass regulatory validation, thereby limiting their widespread deployment in practical applications (Li et al., 2024; Wiśniewska & Tarczyńska, 2022). Therefore, improving model interpretability and optimizing regulatory compatibility is key to the widespread application of food spectroscopic analysis techniques.

#### 3.1.6 Difficulties in equipment integration and lack of uniform standards

Difficulties in equipment integration and the lack of uniform standards have also become one of the critical challenges in the application of food spectroscopic techniques. With the continuous advancement of diverse spectroscopic technologies and instruments, various devices have exhibited differences in accuracy, resolution and operation modes, which have rendered the compatibility and data integration between different devices extremely complex (Fang et al., 2026; Lu, Qin, et al., 2026). In the field of food detection, especially in multimodal spectroscopic analysis, how to effectively integrate data from different spectroscopic instruments has become a major challenge. Devices from different brands have adopted distinct calibration methods, wavelength ranges and resolutions, leading to data heterogeneity, which has further increased the difficulty of cross-device data docking and fusion (Ding et al., 2025; Liu, 2022; Pan et al., 2023).

Moreover, the lack of unified standards and specifications has restricted the interoperability between devices, compromising data consistency and comparability (Zhao, Wang et al., 2025; Zhao, Yu, et al., 2025). To address this issue, existing studies have attempted to map spectral data from different devices to a unified standard by establishing equipment deviation correction models and transfer learning methods, thereby reducing the impact of equipment differences on analytical results (Jiang, Yao, et al., 2023). However, the absence of a universally accepted industry-wide unified standard has limited the popularization and promotion of equipment integration to a certain extent. Therefore, establishing such standards is crucial to unlock the full potential of food spectroscopic techniques, enhancing their accuracy and promoting broader applications.

### 3.2 Future prospects

**Fig. 6:**
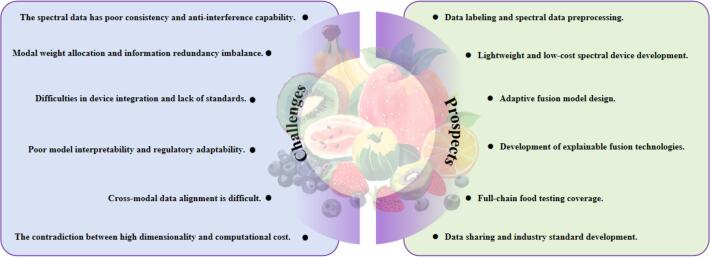
Challenges and prospects of spectroscopy-based multimodal data fusion for food quality analysis.

Given the inherent bottlenecks of spectral data, such as high dimensionality and poor anti-interference capability, upgrading data preprocessing technologies and optimizing device performance can reduce computational costs and mitigate environmental interference while preserving key information. Relying on AI-driven adaptive fusion models and precise alignment strategies helps achieve efficient integration of heterogeneous data and dynamically regulate the contribution of each modality (Huang et al., 2026). In addition, interdisciplinary collaboration, the establishment of unified industry norms, and the development of industry standards will further promote the transformation of technologies from laboratory research to industrial applications (Fig. 6).

#### 3.2.1 Data annotation and spectral data preprocessing

In food spectral analysis, data annotation and spectral data preprocessing are the foundations for ensuring high-quality analytical results. However, with the widespread application of multimodal data in detection, the challenges of data annotation and preprocessing have become more complex (Wu et al., 2025). Spectral data usually contain a large amount of band information, and how to accurately annotate the data of each band and perform screening or dimensionality reduction according to actual conditions has become a key issue. Meanwhile, non-spectral data such as images, videos, and sensor data typically have different dimensions, resolutions, and sampling frequencies, and establishing unified annotation and preprocessing standards for these heterogeneous data remains a major challenge at present. Future research should explore more efficient automated annotation methods, combined with artificial intelligence technologies, to improve the accuracy and efficiency of data annotation without increasing labor costs.

On the other hand, the quality of spectral data preprocessing directly affects the accuracy of subsequent analyses and the robustness of models (Zhao et al., 2016). Regarding preprocessing methods for multimodal data, future research should focus on developing more sophisticated signal denoising and baseline correction techniques, while also considering how to effectively integrate the preprocessing processes of spectral and non-spectral data (Xu et al., 2025). Especially in view of the complementarity between spectral and non-spectral data in multi-source data fusion, preprocessing strategies should be able to balance the processing requirements of data from different modalities, thereby laying the foundation for high-precision and multi-functional food analysis models.

#### 3.2.2 Research and development of lightweight and low-cost spectral devices

The development of portable spectral devices has greatly reduced the operational costs of spectral instruments and effectively addressed the issue of model incompatibility caused by spectral differences between different instruments. Portable HIS devices have been applied to the on-site analysis of meat products, which can meet the demand for rapid detection of meat adulteration. Such devices provide new possibilities for improving the transparency of food traceability and transportation processes, and promote the real-time performance and efficiency of food safety monitoring (Li et al., 2025).

In addition, the development of multispectral integrated portable devices has further reduced the operational costs. A multispectral device that integrates visible light, hyperspectral and microwave radar functions has been developed, with the three functions can operate independently without interfering. This has provided a new idea for the design of multispectral compatible anti-reconnaissance and electrochromic devices (Bertani et al., 2020). This technological breakthrough lays a technical foundation for the application of low-cost, rapid and portable multispectral integrated detection devices in the field of food safety, and is particularly suitable for on-site detection scenarios such as those in developing countries or rural areas.

#### 3.2.3 Adaptive fusion model design

The design of adaptive fusion models plays a crucial role in the field of food analysis, especially in food quality and safety assessment. Through the “modality contribution evaluation module” designed based on the attention mechanism, the model can automatically adjust the weights of different modalities according to the types of food samples (such as liquids and solids), thereby addressing the problem of imbalanced modality weight allocation. This dynamic adjustment method can quantify the contribution of each modality in tasks such as food heavy metal detection, pesticide residue detection, and freshness evaluation, highlight key modal information, suppress interference from redundant or noisy modalities, and further improve the accuracy and robustness of multi-modal fusion models in extracting food quality characteristics, thus supporting efficient and non-destructive intelligent assessment of food quality (Chen et al., 2025).

In addition, combined with the contrastive learning method, spectral and non-spectral data can be mapped to a shared latent space to achieve modal alignment. This method has been applied in the research on monitoring color changes and moisture content during the hot-air drying process of carrot slices. Spectral data were mapped to moisture content, color differences extracted by image analysis, and the spatial distribution of moisture. Analyses were conducted using three chemometric models: PLS, LS-SVM, and back propagation neural network (BPNN). The results showed that the BPNN model exhibited the best performance in moisture content prediction, with a prediction coefficient of determination of 0.991 and an RPD of 11.378 (Liu et al., 2016). Meanwhile, simplified fusion models based on lightweight architectures (e.g., You Only Look Once version 5 (YOLOv5), MobileNet) have laid a foundation for the lightweight development and deployment of detection models in food-related scenarios. For instance, in the task of wheat kernel detection, compared with mainstream lightweight models such as YOLOv5n and YOLOv6n, the YOLO-SDL model not only enhanced detection accuracy but also struck a better balance between parameter count and computational complexity, while retaining efficient real-time detection performance (Qiu et al., 2024).

#### 3.2.4 Development of interpretable fusion technology

To address the black-box problem of the model, the contributions of key spectral bands and non-spectral data are visualized by combining Grad-CAM and Shapley values, and a fusion decision report is generated to meet regulatory requirements (Bessho et al., 2024). The fusion model is divided into a spectral feature extraction module, a non-spectral feature extraction module, and a decision fusion module, each of which can be interpreted independently. The development of interpretable fusion technology is realized through a modular fusion architecture. For the multimodal prototype network applied to cross-domain few-shot hyperspectral image classification, the fusion model is split into a spectral feature extraction module, a non-spectral feature extraction module, and a decision fusion module. By virtue of the collaborative utilization of image spectral information and text semantic information by each module, the model's ability to identify subtle category differences and its classification robustness are enhanced (Liu, Xu, et al., 2025; Wang et al., 2025).

#### 3.2.5 Full-chain food detection coverage

In terms of full-chain food detection coverage, building a multimodal detection system helps achieve synchronous monitoring of the quality of food raw materials, compositional changes during food processing, and quality changes such as food freshness during transportation. Multimodal fusion integrates information collected by different sensors, including cameras, NIRS, thermal imaging, and laser scanning. It enables the simultaneous acquisition of the physical and chemical characteristics of food raw materials, thus realizing more comprehensive and accurate detection (Guo, Lin, Feng, et al., 2025; Xiao et al., 2025). The multimodal detection system, which combined a flexible optoelectronic in-situ sensing system and a multi-input multi-label causal integrated learning model (1DCNN-BiLSTM-ATT), achieved accurate grading of mutton freshness by detecting the impedance data, spectral data and physicochemical indexes of mutton and integrating the 1DCNN-BiLSTM-ATT model (He et al., 2025). During the food circulation process, a cold chain multimodal monitoring terminal has been developed, integrating freshness sensors, temperature sensors and radio frequency identification technology. It enabled real-time tracking of the quality changes of meat products during transportation (Cromwell et al., 2025; Masudin et al., 2021).

#### 3.2.6 Data sharing and industry standard development

To address data security issues, a multi-center federated learning platform has been established. Food enterprises train multimodal models locally and only upload model parameters to the central server, realizing the paradigm of data immobility and model mobility. This approach not only protects data privacy but also enhances the generalization ability of models (Le et al., 2025). In addition, multimodal detection data such as spectral features and physicochemical indicators are uploaded to the blockchain to generate tamper-proof food testing certificates, which consumers can scan to verify via QR codes, thereby improving product credibility (Liu, Xu, et al., 2025). Developing unified industry norms and industrial standards through interdisciplinary collaboration is of great significance for food data sharing and model generalization ability improvement, and further for enhancing food quality control levels. In the future, it is necessary to extensively unite regulatory authorities, scientific research institutions and enterprises to formulate standards for food multimodal fusion detection, clarify various parameters of spectral devices, selection of fusion levels, model verification indicators, and standardize technical applications (Boatwright et al., 2024; Shi et al., 2024).

## 4 Conclusion

Traditional single-modal spectroscopy struggles to meet the food industry's demands for high-speed, real-time, multi-index simultaneous detection, and lacks accuracy and comprehensiveness in complex food quality analysis. This review has summarized the research progress of food spectroscopy and multimodal data fusion, combed the technical characteristics and application scenarios of low-level, mid-level, and high-level fusion, and clarified the core role of multi-source data integration in improving detection performance. The findings showed that coupling spectroscopy with multimodal data fusion significantly enhanced detection accuracy, comprehensiveness, and reliability, providing new insights for food quality and safety control. However, current bottlenecks, including poor spectral data consistency, difficult cross-modal alignment, high equipment costs, and insufficient model interpretability, still restrict industrial applications. Future efforts should focus on collaborative innovation in data preprocessing, equipment/model lightweight, model adaptability, data security/sharing, and industry standard construction. In summary, this review systematically clarified the progress and future directions of this technology, promoting the intelligent and precise development of food detection.

## CRediT authorship contribution statement

**Zhanming Li:** Writing – original draft, Supervision, Funding acquisition. **Wenxuan Deng:** Writing – original draft, Formal analysis, Data curation. **Jing Zhao:** Writing – original draft, Resources, Investigation. **Yan Kong:** Writing – review & editing, Funding acquisition, Conceptualization.

## Declaration of competing interest

The authors declare that they have no known competing financial interests or personal relationships that could have appeared to influence the work reported in this paper.

## Data Availability

No data was used for the research described in the article.
